# Real-time and label-free analysis of binding thermodynamics of carbohydrate-protein interactions on unfixed cancer cell surfaces using a QCM biosensor

**DOI:** 10.1038/srep14066

**Published:** 2015-09-15

**Authors:** Xueming Li, Siyu Song, Qi Shuai, Yihan Pei, Teodor Aastrup, Yuxin Pei, Zhichao Pei

**Affiliations:** 1 College of Science, Northwest A&F University, Yangling, Shaanxi 712100, People’s Republic of China; 2 Clare College, Cambridge CB2 1TL, United Kingdom; 3 Attana, SE-11419, Stockholm, Sweden

## Abstract

A novel approach to the study of binding thermodynamics and kinetics of carbohydrate-protein interactions on unfixed cancer cell surfaces using a quartz crystal microbalance (QCM) biosensor was developed, in which binding events take place at the cell surface, more closely mimicking a biologically relevant environment. In this study, colon adenocarcinoma cells (KM-12) and ovary adenocarcinoma cells (SKOV-3) grew on the optimized polystyrene-coated biosensor chip without fixation. The association and dissociation between the cell surface carbohydrates and a range of lectins, including WGA, Con A, UEA-I, GS-II, PNA and SBA, were monitored in real time and without label for evaluation of cell surface glycosylation. Furthermore, the thermodynamic and kinetic parameters of the interaction between lectins and cell surface glycan were studied, providing detailed information about the interactions, such as the association rate constant, dissociation rate constant, affinity constant, as well as the changes of entropy, enthalpy and Gibbs free energy. This application provides an insight into the cell surface glycosylation and the complex molecular recognition on the intact cell surface, which may have impacts on disease diagnosis and drug discovery.

In the development process of new cancer diagnostic and therapeutic tools, glycobiology has become a new focus due to the various biological functions of membrane glycoproteins and glycolipids on cell surfaces, such as cell recognition, communication, migration, proliferation and death^1^. Abnormal changes in the carbohydrate composition of cancer cell surface have been associated with the survival, invasion and metastasis of cancerous cells^2^. For instance, the metastatic colorectal cancer cells have an elevation in fucosylation in comparison to non-metastatic colorectal cancer cells^3^. Until now, many glycoproteins have been identified as biomarkers for various diseases, such as breast cancer and colorectal cancer^4^. Lectins that can bind to and recognize specific carbohydrate structures have been reported to be important tools for observing glycosylation changes occurring at the surface of cancer cells^5^.

To thoroughly understand these biomolecule recognitions, a wide variety of techniques have been developed for fast and reliable measurements of the interactions, such as X-ray diffraction^6^, nuclear magnetic resonance (NMR)^7^, mass spectroscopy (MS)^8^ and enzyme-linked lectin assays (ELLAs)^9^, as well as fluorescence-based technologies^10^. In contrast to these end-point assays, biosensors based on QCM or surface plasmon resonance (SPR) technologies have proven to be powerful and efficient tools for real-time and label-free monitoring the association and dissociation phases of a complex, enabling binding kinetic studies of biomolecular interactions^11^,^12^,^13^.

Normally, the target molecules to be studied should be isolated and purified from cells, and then were immobilized onto the sensor surface for measuring the pure interaction between a drug candidate and its target^14^. Nevertheless, collection and purification of biomolecules from cells are usually laborious and time consuming. What matters more is that the native environment of the biomolecules is changed and the binding data do not present their native functions in cells accurately, which is particularly problematic for integral membrane proteins that require a lipid bilayer environment to maintain their structure and function^15^. To measure the biomolecule interactions in their native environments directly, recent studies have been concerned with the kinetic evaluation of the biomolecular interactions directly on cell surfaces^3^,^16^,^17^, such as cells grown on a poly-L-lysine coated gold surface to test the binding kinetics of membrane glycoproteins based on SPR technologies^18^. Previous studies have also utilized QCM cell biosensors to monitor protein-carbohydrate interactions in real-time by employing cancer cells grown on a polystyrene coated surface^3^,^17^ where cells on the sensor surface normally need to be fixed by using standard formaldehyde-based procedures. However, the study for real-time analysis of biomolecular interactions directly on unfixed cell surfaces by using QCM biosensor have not been reported.

Furthermore it is increasingly acknowledged that a more complete understanding of the interaction of biological macromolecules requires not only the kinetic information but also the thermodynamic properties, which is also essential for the development of new pharmaceutical substances for cancer diagnosis and therapeutics^19^,^20^. Isothermal titration calorimetry (ITC) is a classical method for thermodynamic analysis that directly measures the heat released or absorbed upon molecular interactions to estimate the thermodynamic properties^21^. However, it requires a substantial amount of the interaction partners^22^, and the extremely low concentrations of membrane receptors present in biological tissues make it very difficult to obtain sufficient amount of samples, resulting in many microcalorimetric determinations of thermodynamic parameters impossible, which greatly limits its application in membrane receptors studies. Recently, biosensor technology has been successfully applied to obtain the thermodynamic parameters of biomolecule interactions by measuring affinity constant (*K*_D_) over a range of temperatures, combined with van’t Hoff plot analysis^23^. For example, Day *et al.* and de Mol *et al.* obtained the thermodynamic information of molecule interactions by measuring interactions at different temperatures with SPR biosensors, which features the advantages of the relatively low consumption of samples and simultaneous collection of kinetic data^24^,^25^. In addition, the biosensor method also allows a transition state analysis of the binding process from the kinetic data according to Eyring’s transition state theory^25^. This methodology give interesting complementary information to ITC, since only the physical association and dissociation of the two molecular species are monitored, whereas calorimetric assays the total heat effects during the interaction, including heat of dilution or mixing^26^. In particular, biosensors can detect very low concentrations of analytes, enabling the direct detection of biomolecular interactions on cell surfaces^3^,^16^,^17^,^18^. However, thus far, there are no reports combining the thermodynamic and kinetic analysis of the biomolecule interactions on intact cell surfaces.

In this study, we present a novel approach for real-time and label-free analysis of binding thermodynamics and kinetics of carbohydrate-protein interactions on intact cell surfaces using a QCM biosensor, where cancer cells are grown on an optimized polystyrene-coated biosensor chip without fixation. To the best of our knowledge, this is the first report in which the biosensor was fabricated and exploited for evaluating the binding thermodynamics and kinetics of the biomolecule interactions on intact cell surfaces.

## Results and Discussion

### Stability, reproducibility, and specificity of the un-fixed cell sensor surface

**Figure 1:**
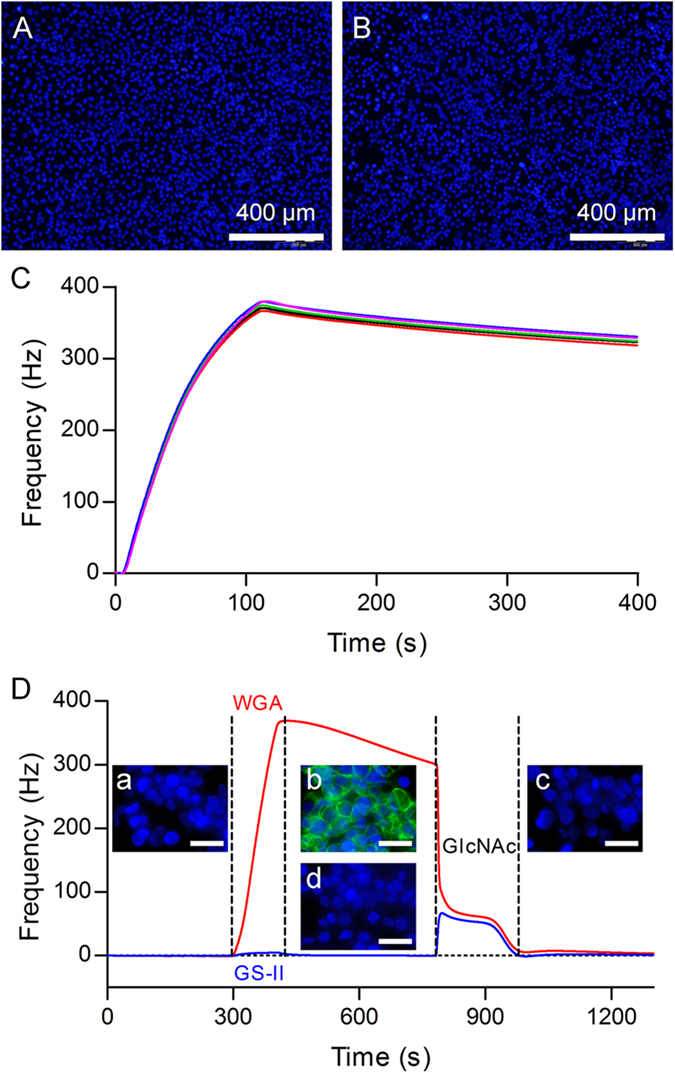
Stability, reproducibility, and specificity of the unfixed cell sensor surface. Cell coverage on the sensor surface was evaluated before (**A**) and after (**B**) QCM measurements by staining the nuclei of SKOV-3 cells with Hoechst 33342 and visualizing under a fluorescent microscope (Olympus BX53). (**C**) Reproducibility of 5 cycles of the interaction between WGA (50 μg/mL) and unfixed SKOV-3 cells on sensor surface with regeneration step (2 injections of 300 mM GlcNAc) in between. (**D**) The bindings of WGA-FITC (red line) and Alexa Fluor® 488 conjugated GS-II (blue line) with SKOV-3 cells were evaluate by QCM measurements and microscopic evaluation. Fluorescent images of nuclei (in blue) and lectin (in green) were taken before (a) and after (b) WGA-FITC; (d) Alexa Fluor® 488 conjugated GS-II injection of lectin, as well as after regeneration (c). The scale bars stand for 40 μm.

In the previous reports^3^,^17^, adherent cells were grown on a chip surface and fixed in formaldehyde. Formaldehyde-based fixation cross-links cell surface protein, therefore, the formaldehyde fixation process may impact the recognition of cell surface epitopes compared with the intact cells. In our study, we found that cells with strong adherent properties could grow on the optimized polystyrene-coated biosensor chip without fixation for analysis of the biomolecule interactions on intact cell surfaces. To evaluate the stability of cells on the sensor surface without fixation, SKOV-3 cells were seeded and grown on the sensor surface. Cell coverage on the sensor surface was evaluated before and after QCM measurements by staining the nuclei with Hoechst 33342 and visualizing under a fluorescent microscope (Olympus BX53). As shown in Fig. 1A,B, most cells remained on the sensor surface after several injection of lectins and regeneration solutions at different temperatures, which indicated the capability of performing interaction studies on the sensor surface without fixation. To evaluate the reproducibility of interactions on the unfixed cell surface, the measurement of the interaction between wheat germ agglutinin (WGA) and SKOV-3 cells was performed 5 cycles with the regeneration step (2 injections of 300 mM N-acetylglucosamine, GlcNAc) in between. Figure 1C shows good reproducibility of the interactions. To demonstrate the frequency shift monitored on the sensorgram specifically reflected the interactions occurring at the cell surface between lectin and membrane glycoconjugates, a microscopic evaluation of lectin-cell binding was performed. As shown in Fig. 1D, only cell nucleuses were observed before lectin injection (a), confirming the presence of cells on the surface. After the injection of fluorescein isothiocyanate conjugated wheat germ agglutinin (WGA-FITC), the interaction with cells was monitored by QCM biosensor (red line). Green fluorescence was also observed and showed a mainly peripheral localization of the lectin (b). After an injection of 300 mM GlcNAc, the frequency restored to the base line and the green fluorescence disappeared (c), demonstrating complete remove of the WGA-FITC from the cell surface. However, no green fluorescence was observed after the injection of Alexa Fluor® 488 conjugated Griffonia Simplicifolia Lectin II (GS-II) (d), which had a very low response on the sensorgram. These observations indicated that the frequency shift monitored by QCM biosensor specifically reflected the interactions between lectin and membrane glycoconjugates occurring at the unfixed cell surface.

### Lectin screening

**Figure 2:**
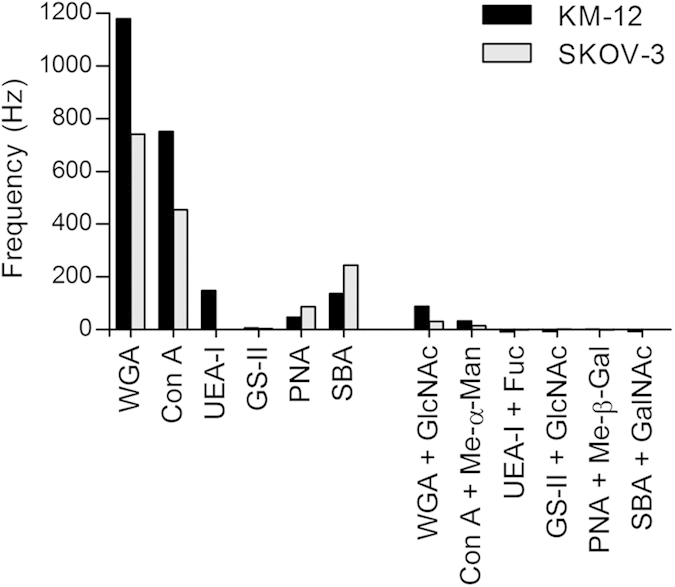
Lectin screening. WGA, Con A, UEA-I, GS-II, PNA and SBA at 0.96 μM were injected over the KM-12 and SKOV-3 cell surfaces. The maximum frequency shift obtained for each lectin was recorded and summarized. Control experiments were performed by pre-incubation of lectins with 300 mM corresponding ligand for 30 min. The inhibition of lectin binding illustrated the specificity of lectin binding to carbohydrate residues. Me-α-Man: α-methylmannoside; and Me-β-Gal: β-methylgalactoside.

To evaluate the glycosylation of KM-12 and SKOV-3 cell surface, real-time lectin screening of a range of lectins was performed. Lectins, including WGA, Concanavalin A (Con A), ulex europaeus agglutinin I (UEA-I), GS-II, peanut agglutinin (PNA) and soybean agglutinin (SBA), which can specifically recognize and bind to GlcNAc/sialic acid, α-mannoside (Man)/α-glucoside (Glc), L-Fucose (Fuc), GlcNAc, β-galactoside (Gal), and N-acetylgalactosamine (GalNAc) respectively, were sequentially injected over the cell surfaces, separated by regeneration steps in between. The responses were simultaneously monitored by the Attester software in real time and the maximal frequency shifts obtained for each lectin were recorded. As can be seen in Fig. 2, the different frequency shifts of diverse lectins produced from the interactions between lectins and cancer cell surfaces indicated the different glycosylation on cell surfaces. WGA, which bound to sialic acid and GlcNAc residues, exhibited high binding to the SKOV-3 cells (741 Hz) and very high binding to the KM-12 cells (1180 Hz), which could be explained by the binding of WGA to highly branched glycoconjugates at the surface of KM-12 cells. However, GS-II, which bound high selectivity to terminal GlcNAc residues, exhibited very low binding to these two cells (5.5 Hz and 2.6 Hz, respectively). These results indicated that SKOV-3 and KM-12 cell surfaces had a very low level expression of GlcNAc, but a very high level of sialic acid expressions. The binding frequencies of Con A, SBA, PNA and UEA-I to KM-12 cell surface were recorded with maximum frequency shifts of 751 Hz, 136 Hz, 47 Hz and 147 Hz, respectively. The binding frequencies of Con A, SBA and PNA to the SKOV-3 cell surface were recorded with maximum frequency shifts of 454 Hz, 244 Hz and 86 Hz, respectively, whereas no responses were produced from UEA-I. The results showed that the response varied considerably depending on lectins and cells, which suggested that these two cells expressed different levels of Man-/Glc-, GalNAc-, Gal- and Fuc-containing glycoconjugates on their surfaces. Control experiments were performed through blocking the binding sites of the lectins, where lectins were pre-incubated with corresponding ligand and injected over the sensor chip (referencing with the corresponding ligand-containing solution). The inhibition of lectin binding was observed (Fig. 2) thereby illustrating the specificity of lectin binding to carbohydrate residues on cell surface.

### Temperature dependence of kinetics and affinity

**Figure 3:**
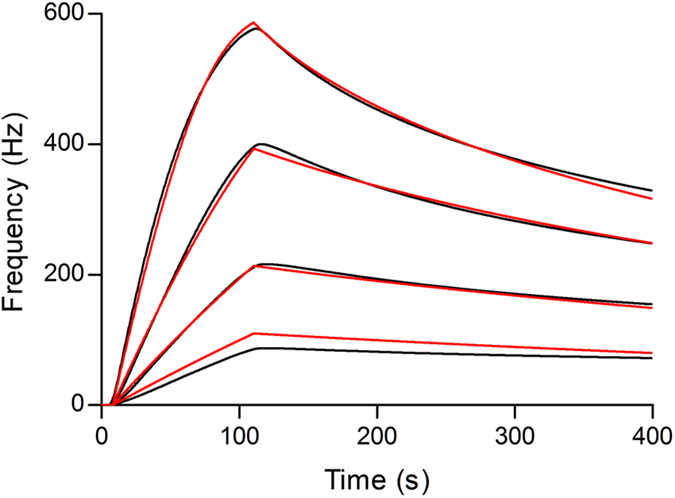
Kinetic evaluation of the interactions between WGA and KM-12 cells at 28 °C. WGA at 12.5–100 μg/mL was injected over the cell surface and the responses were recorded (black lines). Theoretical 1:1 fit generated by the Evaluation software (Attana) were overlaid (red lines). The association and dissociation rate constant (*k*_ass_ = 5.45 × 10^4^ M^−1^s^−1^, *k*_diss_ = 5.09 × 10^−3^ s^−1^) as well as the affinity constant (*K*_D_ = 93.4 nM) were obtained.

The temperature dependence of the kinetics and affinity of the interaction between WGA and KM-12 cell surface glycan was determined at 7, 14, 21, 28 and 35 °C, respectively. Figure 3 shows a typical example of the frequency shifts recorded from the interaction at 28 °C along with the theoretical 1:1 fit produced by Evaluation software (Attana). The kinetic parameters of the interaction between WGA and KM-12 cell surface carbohydrates were determined by injecting a serial dilution concentration series of WGA in running buffer ranging from 12.5 to 100 μg/mL over the sensor surface with regeneration steps in between. The sensorgram obtained was analyzed using Evaluation software provided with Attana Cell A200 instrument, which interprets the kinetics of a reaction by fitting theoretical models based on hypothetical mechanisms. A 1:1 interaction model with mass transport limitation was used to global fit the binding curves, and the kinetic rate constants, such as association rate constant (*k*_ass_ = 5.45 × 10^4^ M^−1^s^−1^) and dissociation rate constant (*k*_diss_ = 5.09 × 10^−3^ s^−1^), were obtained. From these values, the affinity constant (*K*_D_ = 93.4 nM) was calculated by the ratio of binding rate constants *k*_diss_/*k*_ass_. Moreover, the kinetics and affinity of the WGA and KM-12 cell interactions at 7, 14, 21 and 35 °C were also evaluated respectively, and the *k*_ass_, *k*_diss_, as well as *K*_D_, were obtained for each temperature. The resulting data was presented in Table 1, and as can be seen, the association rate increased 5.4-fold in going from 7 to 35 °C, while the dissociation rate increased 43-fold, resulting in a 8-fold loss of affinity from 22.3 to 179 nM. In addition, the temperature dependence of the kinetics and affinity of the interaction between UEA-I and KM-12 cells, as well as WGA and SKOV-3 cells, was evaluated and the data was shown in Table 1. These results indicated that both the association and dissociation rates increase with temperature, but the effect of dissociation is much more significant.

### Thermodynamic studies

**Figure 4:**
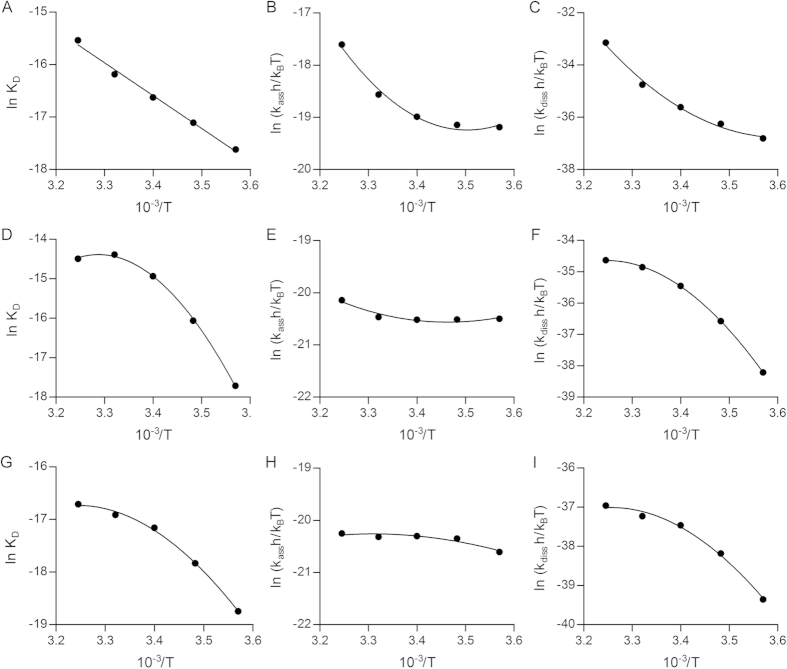
van’t Hoff plots (A,D and G) and Eyring plots (B,C,E,F,H and I) of the interaction between lectins and cells. (**A–C**) WGA and KM-12 cells; (**D–E**) UEA-I and KM-12 cells; and (**G–H**) WGA and SKOV-3 cells.

Accurate information of the thermodynamic parameters, for example if a protein-protein interaction is enthalpy or entropy driven, has already been proven to be useful in pharmaceutical research^19^,^20^. In this study, the interaction of WGA with KM-12 cell surface carbohydrates was used as a model system for the thermodynamic analysis of biomolecule interaction occurring on cell surface by QCM biosensor. The affinity constant (*K*_D_) of the interaction between WGA and KM-12 cells was obtained at different temperatures as described above. The van’t Hoff plot of ln *K*_D_ vs 1/T is shown in Fig. 4A. It appears that the plot is nearly linear, indicating that Δ*H*° and Δ*S*° were practically independent of temperature. A linear regression analysis of these data to van’t Hoff equation (Eq. 2)^25^,^27^ using GraphPad Prism 5 software was performed (Fig. 4A) giving the following thermodynamic parameters of the interaction at standard temperature: 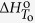 = −52.1 kJ mol^−1^, 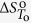 = −39.1 J K^−1^ mol^−1^ and 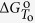 = −40.4 kJ mol^−1^ (Table 2). These preliminary data indicates that the interactions between WGA and KM-12 cell surface carbohydrates are predominantly enthalpy driven. Furthermore, the thermodynamic parameters of the interaction between UEA-I and KM-12 cells, as well as WGA and SKOV-3 cells, were obtained by nonlinear regression analysis with integrated van’t Hoff equation (Eq. 1) (Fig. 4D,G). The obtained thermodynamic parameters (Table 2) suggested that the interactions between UEA-I and KM-12 cells are predominantly enthalpy driven and the interaction between WGA and SKOV-3 cells is driven by both entropy and enthalpy changes.


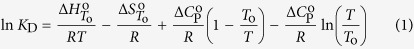



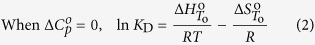


Where *K*_D_ is the affinity constant, 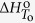
is the standard enthalpy change at reference temperature, 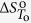
is the standard entropy change at reference temperature, *R* is the universal gas constant, *T* is the absolute temperature (K), *T*_o_ is the reference temperature (298.15 K for standard conditions), 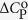 is the heat capacity change under standard conditions. (The superscript ‘o’ denotes the standard state).

In addition, determination of the temperature dependence of *k*_ass_ and *k*_diss_ allows thermodynamic analysis of the activation for association and dissociation, via fitting the data to the Eyring equation (Eq. 3). As shown in Fig. 4B,C,E, the plots for *k*_ass_ and *k*_diss_ are concave, indicating a positive heat capacity change (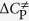) value for the formation of the transition state from the reactants or complex. On the contrary, Fig. 4F,H,I indicate a negative heat capacity change. The thermodynamic parameters for activation were obtained from fitting the data to Eq. 3 25 and are shown in Table 2. This information can be used to break down the free energy barriers into their enthalpy and entropy components.


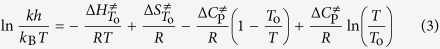


Where *k* is the kinetic rate constant for the interaction in the corresponding direction (*k*_ass_ or *k*_diss_), *k*_B_ is Boltzmann’s constant (1.381 × 10^−23^ J K^−1^) and *h* is Plank’s constant (6.626 × 10^−34^ J s), ≠ stands for the formation of the transition state for the forward or back reaction, 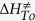 and 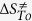 is the activation enthalpy and entropy at the reference temperature, respectively. 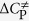
is the heat capacity change under standard conditions.

In conclusion, we have developed a novel approach based on QCM technology to evaluate the binding thermodynamics of carbohydrate-protein interactions on intact cell surfaces, more closely mimicking a biologically relevant environment. Cancer cells grew on the sensor chip surface without fixation, and the unfixed cancer cell surface features good stability, reproducibility, and specificity for lectin-cell interactions. Surface glycosylation of cancer cells was evaluated by real time detection of the interaction between cell surface glycan and a range of lectins. The temperature dependence of kinetics and affinity of the interaction between lectins and cell surface glycan was detected to show that the association and dissociation rates as well as the affinity constant of the interaction increase significantly with the temperature. The thermodynamic parameters of the interaction between lectins and cell surface glycan indicate that the interaction of WGA with KM-12 cells and UEA-I with KM-12 cells are predominantly enthalpy driven, and the interaction of WGA with SKOV-3 cells is driven by both entropy and enthalpy changes. This approach allows us to directly determine the thermodynamics of the biomolecule interactions on intact cell surface, which may assist the study of the biological activities of glycoproteins and the discovery of drugs, as well as the diagnosis of diseases.

## Methods

### Chemicals and reagents

Con A, WGA-FITC, Hoechst 33342 and glycine were purchased from Sigma. WGA, UEA-I, PNA and SBA were purchased from Vector Laboratories. GS-II was purchased from Molecular Probes. The human colon adenocarcinoma cells (KM-12) were purchased from Guangzhou Jennio Biotech Co.,Ltd (China) and the human ovary adenocarcinoma cells (SKOV-3) were kindly provided by Attana AB (Stockholm, Sweden). All biosensor experiments were undertaken using an Attana Cell A200 QCM instrument and the running buffer was phosphate buffered saline (PBS).

### Cell culture

KM-12 and SKOV-3 cells were cultured in RPMI 1640 medium (Gibco) supplemented with 10% (v/v) fetal bovine serum and 1% (v/v) penicillin/streptomycin. All the cell lines were cultured at 37 ˚C with 5% CO_2_ and 95% humidity, and were maintained by changing the medium every 2 days and passaging using standard trypsinization protocols when they were approximately 75% confluent.

### Preparation of cell-biosensor chips

The cell-biosensor chips were prepared according to the literatures^3^,^17^. KM-12 or SKOV-3 cells were grown directly on the cell optimized polystyrene (COP-1) QCM sensor chip surfaces (Attana AB, Stockholm, Sweden) for evaluation in the QCM biosensor. In this study, 80 000 cells were seeded to the cell sensor chip and cultured for 24 h at 37 °C, allowing the cells adhere, grow and proliferate on the surface. The cell coverage on the sensor surface was evaluated by staining the nuclei with Hoechst 33342 and visualizing under a fluorescent microscopy (Olympus BX53). Then the chip was docked into an Attana Cell A200 QCM instrument and a biosensor experiment measuring the interactions between lectins and the cells can be started.

### QCM analysis of lectin-cell interactions

All interaction measurements were performed using an Attana Cell A200 QCM biosensor and the running buffer was PBS in a temperature range of 7 to 35 °C. The prepared cell chips were docked into the instrument and stabilized under a continuous flow (20 μL/min) of running buffer. The measurements were initiated when the resonant frequency was stable (baseline drift <0.2 Hz/min). Lectin samples prepared in running buffer were injected over the surface of chips, monitoring the association for 105 s and dissociation for 295 s. The resonant frequency of the quartz crystal and the frequency shift (Δ*f*) associated with association or dissociation were recorded with the Attester software in real time. Following each association and dissociation cycle, the cell chips were regenerated between measurements by 1–2 injections of 10 mM glycine pH 2.0 or 300 mM corresponding monosaccharides to dissociate the bound lectins, and immediately re-equilibrated with running buffer.

## Additional Information

**How to cite this article**: Li, X. *et al.* Real-time and label-free analysis of binding thermodynamics of carbohydrate-protein interactions on unfixed cancer cell surfaces using a QCM biosensor. *Sci. Rep.* **5**, 14066; doi: 10.1038/srep14066 (2015).

## Figures and Tables

**Table 1:** Temperature-dependence of kinetics and affinity of the interaction between lectins and cells.

*t* (˚C)	*k*_ass_ (10^4^ M^−1^ S^−1^)	*k*_diss_ (10^−4^ S^−1^)	*K*_D_ (nM)
WGA and KM-12			
7	2.71	6.05	22.3
14	2.90	10.8	37.2
21	3.47	20.9	60.1
28	5.45	50.9	93.4
35	14.5	259	179
UEA-I and KM-12			
7	0.73	1.48	20.3
14	0.74	7.82	106
21	0.75	24.6	327
28	0.81	45.7	564
35	1.15	58.4	508
WGA and SKOV-3			
7	0.66	0.47	7.22
14	0.87	1.57	18.0
21	0.93	3.30	35.4
28	0.94	4.26	45.2
35	1.03	5.71	55.5

**Table 2:** Thermodynamics parameters for the interaction of WGA and KM-12 cells.

thermodynamics parameters	interaction	activation to transition state	
		association	dissociation
WGA and KM-12			
Δ*H*/kJ mol^−1^	−52.1 ± 2.5	58.7 ± 5.1	114.4 ± 7.9
Δ*S*/J K^−1^ mol^−1^	−39.1 ± 8.7	41.2 ± 16.9	92.0 ± 26.1
Δ*C*_p_/kJ K^−1^ mol^−1^	–	4.6 ± 0.8	5.4 ± 1.3
ΔG/kJ mol^−1^	−40.4	46.4	86.9
UEA-I and KM-12			
Δ*H*/kJ mol^−1^	−48.1 ± 2.5	14.7 ± 3.1	62.9 ± 0.9
Δ*S*/J K^−1^ mol^−1^	−40.2 ± 8.2	−120.7 ± 10.4	−80.6 ± 3.0
Δ*C*_p_/kJ K^−1^ mol^−1^	8.0 ± 0.4	1.6 ± 0.5	−6.4 ± 0.1
ΔG/kJ mol^−1^	−36.2	50.7	86.9
WGA and SKOV-3			
Δ*H*/kJ mol^−1^	−35.7 ± 2.8	3.67 ± 3.0	39.4 ± 5.2
Δ*S*/J K^−1^ mol^−1^	21.3 ± 9.5	−156.2 ± 10.0	−177.5 ± 17.2
Δ*C*_p_/kJ K^−1^ mol^−1^	3.5 ± 0.5	−0.91 ± 0.49	−4.4 ± 0.8
ΔG/kJ mol^−1^	−42.1	50.2	92.3
